# The Research Council System and the Politics of Medical and Agricultural Research for the British Colonial Empire, 1940–52

**DOI:** 10.1017/mdh.2013.17

**Published:** 2013-07

**Authors:** Sabine Clarke

**Affiliations:** Department Of History, The University of York, Heslington, York YO10 5DD UK

**Keywords:** Medical Research Council, Agricultural Research Council, Colonial Office, 1940 CDW Act, Colonial Medical Research Council, East Africa

## Abstract

Historical accounts of colonial science and medicine have failed to engage with the Colonial Office’s shift in focus towards the support of research after 1940. A large new fund was created in 1940 to expand activities in the colonies described as fundamental research. With this new funding came a qualitative shift in the type of personnel and activity sought for colonial development and, as a result, a diverse group of medical and technical officers existed in Britain’s colonies by the 1950s. The fact that such variety existed amongst British officers in terms of their qualifications, institutional locations and also their relationships with colonial and metropolitan governments makes the use of the term ‘expert’ in much existing historical scholarship on scientific and medical aspects of empire problematic. This article will consider how the Colonial Office achieved this expansion of research activities and personnel after 1940. Specifically, it will focus on the reasons officials sought to engage individuals drawn from the British research councils to administer this work and the consequences of their involvement for the new apparatus established for colonial research after 1940. An understanding of the implications of the application of the research council system to the Colonial Empire requires engagement with the ideology promoted by the Agricultural Research Council (ARC) and Medical Research Council (MRC) which placed emphasis on the distinct and higher status of fundamental research and which privileged freedom for researchers.

## Introduction

Our existing accounts of colonial science and medicine in the twentieth century have failed to grasp the extent to which the Colonial Development and Welfare (CDW) Act of 1940 represented a major turning point in the history of the British Colonial Empire. There is a tendency in accounts of the medical services in the British Colonial Empire to discuss the post-war period superficially, if at all.^1^ Those accounts of colonial science and medicine that do consider the late colonial period often fail to engage with the Colonial Office’s shift in focus towards the support of research after 1940.^2^ A large new fund was created in 1940 to expand activities in the colonies described as fundamental research. With this new funding came a qualitative shift in the type of personnel and activity sought for colonial development; the goal was not to supplement the existing work of medical or scientific departments in the colonies. As a result, a diverse group of medical and technical officers existed in Britain’s colonies by the 1950s that included doctors, medical researchers, agricultural extension officers, plant breeders, entomologists and a range of other grades and specialists. Around forty new institutions had been created across the Colonial Empire for the work of research staff and these individuals were recruited to a new Colonial Research Service, separate from the existing technical services. The fact that such variety existed amongst British officers in terms of their qualifications, institutional locations and also their relationships with colonial and metropolitan governments makes the use of the term ‘expert’ in much existing historical scholarship on scientific and medical aspects of empire problematic.

The work of historians such as Christophe Bonneuil and Joseph Hodge have done much to elucidate the role of agricultural officers who worked on development schemes in Africa after 1940.^3^ We should not take these descriptions to be general accounts of all agricultural expertise in the colonies as this literature is solely focussed on the work of members of the Colonial Agricultural Service. Missing in our current literature is recognition that the understandings we have of the role of agricultural or medical officers in the colonies after 1940 are not necessarily applicable to the work of researchers in these fields. The two types of officer differed in terms of their relationship with the imperial and colonial state, other colonial officers and local African communities. The new arrangements for research created at the Colonial Office in the early 1940s aimed to give medical and agricultural research staff a degree of autonomy with regard to colonial administrations, and this along with the creation of a separate research service was intended to confer upon individuals with specialist qualifications such as biochemists and entomologists a greater status than they had previously enjoyed. Our accounts of colonial science and medicine need to pay greater attention to the distinction made between research and other activities falling under the heading of scientific and medical work as this distinction was crucial for the individuals who organised colonial research after 1940.

This paper will describe the new arrangements that were introduced to accomplish the substantial expansion of research in Britain’s colonies after 1940 and consider the reasons for the eventual form of these arrangements. The argument that will be made is that an understanding of the novel machinery introduced for colonial medical and agricultural research after 1940 requires an appreciation of developments in the organisation of research at home. The research apparatus introduced for the Colonial Empire was informed by a liberal ideology of research that had become institutionalised in Britain with the rise of the research council system. This ideology promoted the claim that research could only really flourish if conditions were provided for individuals in which they had freedom to work. Freedom in this case meant the absence of interference from those who lacked experience of carrying out research themselves. This could include politicians, civil servants and even scientific, medical and technical staff, if their work was not judged to involve research. This rhetoric worked to enhance the standing of the research councils. The Agricultural Research Council (ARC) and Medical Research Council (MRC) claimed that, unlike government departments at home and in the colonies, research councils were bodies governed by experienced researchers and therefore only they were qualified to administer research. The notion that the research councils were the true bastions of research in Britain was taken to its extreme by the MRC who worked to subsume all medical research in Britain within its control.

When the Colonial Office came to formulate plans for a substantial expansion of agricultural and medical research for the colonies in the 1940s it turned to the Agricultural Research Council and the Medical Research Council. Officials at the Colonial Office did this because they believed they were moving into a new area of administration where advice would be necessary but also because the involvement of representatives of the research councils was considered essential in order to raise the prestige of colonial research so the Colonial Office could attract high-flying research staff. By 1947 the Colonial Office had created ten new committees in London to administer money from the research allocation of the CDW Acts.^4^ The Committee for Colonial Agricultural, Animal Health and Forestry Research or CCAAHFR was appointed to oversee agricultural research across the Colonial Empire and included members of the ARC. In the case of medical research, a Colonial Medical Research Committee was formed of which half the members were drawn from the MRC. Both committees used the work of the research councils at home as a model, and a justification, for the new arrangements they wished to introduce for the colonies between 1945 and 1952.

This assertion of metropolitan expertise when it came to the introduction of a substantial expansion in research in Britain’s colonies after 1940 is not recognised in the existing literature. It was a deviation from the trend that had been occurring before 1940. In East Africa plans had been formulated during the late 1930s for the creation of new regional bodies to co-ordinate research in medicine, agriculture and veterinary science. The initiative for the Standing Medical Research Committee proposed in 1936 came from doctors and entomologists based in Tanganyika, Kenya and Uganda and reflected a desire for better organisation and funding that had arisen at the colony level.^5^ This trajectory in which members of the Colonial Medical Service based in East Africa determined the direction of medical research in the region was disturbed in 1945 with the formation of the Colonial Medical Research Committee. The plans conceived by the CMRC between 1945 and 1952 worked to marginalise the input of doctors and scientists based in East Africa and other parts of the British Empire in the planning and execution of medical research. With the creation of new research committees at the Colonial Office after 1940 the Colonial Office privileged expertise in the administration of research over the place-based knowledge held by existing members of the medical and scientific services.^6^

As the 1940s progressed, the Colonial Office had to balance a number of competing concerns. Initially the priority of the Colonial Office was the recruitment of highly capable research staff to work on the expanding range of research schemes. During the course of the second half of the 1940s other issues came to the fore, however, including the need to appreciate rising political consciousness amongst colonial peoples. By the early 1950s the Colonial Office was attempting to place limits on the autonomy and power held by the CMRC on the grounds that representatives of the MRC were too ignorant of the changing political conditions in Britain’s colonies to be allowed sole responsibility for medical research.

## The Rise of Research

The period between World War I and 1950 saw scientific research move from being an area that was said to be neglected in Britain to a field of intense state interest.^7^ The narrative of state-funded science in Britain that has emerged through the works of historians such as Peter Alter describe how the creation of the Department of Scientific and Industrial Research or DSIR marked the beginning of considerably expanded state commitment to research.^8^  The DSIR  was followed by the Medical Research Council (MRC) in 1919 and Agricultural Research Council (ARC) in 1931 forming the British Research Council system in which state funds were provided for research into medical, agricultural and industrial issues in universities and research units.^9^ It was claimed at the time that the triumph of this system resided in the fact that it provided government funding for science without government interference since the research councils were not headed by ministers but reported to parliament through the Privy Council. The virtue of this arrangement was said to be that administrators or politicians did not direct the DSIR, MRC and ARC in their choice of research programmes but these bodies made their own research decisions through an advisory council of scientists that headed each body. A distinction was made between this arrangement and that which existed in government departments with the concomitant claim that the science prosecuted by these departments was not proper research but more often short-term, problem-solving enquiries.^10^  Freedom from departmental  direction was a source of pride for the research councils.^11^  The MRC claimed that, whilst the council was in receipt of state funds it was not subject to government control. Edward Mellanby, who was appointed Secretary in 1933, stated that this independence of the MRC from direct departmental supervision and political influence was key ‘to the Council’s reputation as the purveyor of truth’.^12^

From this narrative we might come to the conclusion that research in Britain was the sole preserve of the universities and research councils. In historical accounts of the period between 1914 and 1950 the science undertaken by government departments has in fact often been overlooked. One exception is the work of David Edgerton which has done much to transform this picture of the rise of state-funded science in the first half of the twentieth century by showing that spending on research by the Service Departments – the Admiralty, Air Ministry and Navy – was in excess of that of the civil research councils.^13^

In addition to recognition of the large proportion of state funds that were spent on research by military departments, we need to adjust our narrative of the rise of research in Britain by recognition of the very large commitment to colonial research from 1940 onwards by another government department, the Colonial Office. The passing of the 1940 Colonial Development and Welfare Act created a Research Fund of 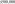 pa, which was then increased to  million with renewal of the Act in 1945. In comparison to previous provision this was Britain’s greatest ever commitment to scientific research for the Colonial Empire. It was also a fund that gave the Colonial Office a greater allocation for research than either the MRC or ARC until 1950.^14^ Only the DSIR commanded a larger fund for scientific research in the civil sphere and this was a body entirely devoted to supporting research. The 1940 Act elevated the Colonial Office to the level of a fourth research council in Britain, except that it was in fact a government department. With the creation of the Research Fund in 1940 the Colonial Office was faced with the issue of how to recruit large numbers of well-qualified research staff. Officials believed that they would struggle to attract the best scientific researchers because the status of scientific work done by government departments in Britain was low.^15^

The resolution to this problem was the appointment of a number of committees at the Colonial Office that were populated by eminent scientists, many of whom were associated with Britain’s research councils.^16^ The first of these was the Colonial Research Committee created in 1942 to provide oversight of the full range of colonial research. This committee included a number of prominent individuals such as A.V. Hill, the Secretary of the Royal Society, Edward Mellanby the Secretary of the MRC, W.C.C. Topley of the ARC and Alexander Carr-Saunders, the Director of the London School of Economics (LSE).^17^ The aim was to create a body capable of commanding the respect of British scientists and to confer upon colonial research a status comparable with the existing research councils in Britain.^18^ In addition to the raising of the prestige of colonial research, the invitation to members of the domestic research councils to sit on the CRC and other committees created to administer research had a practical value, according to the Colonial Office’s Adviser on Animal Health, since, ‘They control, in their various spheres, all research in the UK which is financed from public funds. Their secretaries are in touch with all workers’.^19^ The appointment of representatives of the research councils was necessary for two reasons: to raise the standing of the research work done through the Colonial Office and to gain access to researchers.

## The Formation of Agricultural Research Institutions after 1940

In June 1945 the Colonial Office formed a new committee to oversee the expansion of research in agriculture, veterinary science and forestry. This was the Committee for Colonial Agricultural, Animal Health and Forestry Research or CCAAHFR. As with the other research committees that were formed at the Colonial Office between 1942 and 1947, the CCAAHFR contained members drawn from the domestic research councils, in this case John Fryer, Frank Engledow, John Simonsen and John Smith from the ARC.^20^ The formation of a number of sub-committees dealing with research into cocoa, soils and stored products drew even greater numbers of scientists from the ARC and British agricultural research establishments into discussions about colonial agriculture.

The new committee for agricultural research differed from the pre-existing body that provided the Colonial Office advice on agricultural policy, the Colonial Advisory Council of Agriculture, Animal Health and Forestry.^21^ The CCAAHFR had greater authority than this older body, which was restricted to providing comment on agricultural plans conceived in the colonies or by officials in London. The CCAAHFR was not merely advisory, but had executive functions so that it determined the new arrangements to be introduced for research in the colonies and had the power to initiate research projects.

Colonial Office officials agreed with the scientists they had recruited on the need to use the research fund to introduce a comprehensive array of programmes of fundamental research. Fundamental research was defined as long-range studies of widespread and basic problems and the acquisition of knowledge through this work about conditions in Africa, for example, was said to be crucial to the successful development of Britain’s colonies after 1940. Officials supported the claim by members of its new research committees that, for fundamental research to prosper, research workers must be given a degree of independence from the existing colonial administrations. In 1946 Charles Carstairs wrote to the Central African Council to explain that the intention of the Office in promoting fundamental research was to support ‘long term work bearing on the basic problems of an area, best conducted on a semi-independent basis’.^22^ Officials endorsed the claim that arrangements were necessary to ensure academic freedom for researchers, said to be a prerequisite for good research work and an important condition if the Colonial Office was to attract proficient research workers. The scientist E.B. Worthington had said in a comprehensive study of science in Africa published in 1938 that research had failed to thrive in the colonies in the past because there were few individuals in the existing technical services who fully understood its necessity and the conditions needed for its successful prosecution. According to Worthington, too often senior officers in the Colonial Agricultural Service directed their most capable staff to work on mundane, routine work.^23^ In order for research to flourish it would be necessary to create new arrangements in which research was removed from the ambit of staff with little experience of research and placed under new heads of research in separate departments.^24^

The notion of freedom for researchers, and also the committees in London that oversaw their work, was explained in a Colonial Office note to the Conference of African Governors of 1947:

It implies on the one hand some degree of independence of local administrations, except for disciplinary purposes, and on the other hand scientific control by scientists of assured reputation. The Secretary of State has sought to meet these requirements by giving to the appropriate Research Committee a special position of independence. While nominally the Committees are advisory, he has undertaken to be guided by their advice in scientific control of research workers in all save the most exceptional of circumstances.^25^

Colonial governments were not only expected to accept that their research needs were to be overseen by committees based in London, they were also expected to agree to the creation of research laboratories in their territories over which they would have very little control.

With the creation of the CCAAHFR the Colonial Office endorsed an approach to organising agricultural research that set out to reproduce in the colonial context the type of arrangements that existed for agricultural research at home under the ARC. The focus of the CCAAHFR was on the formation of new research institutions that would operate with some autonomy from the existing Departments of Agriculture in the colonies. It was agreed that new laboratories in the colonies should be created that served groups of colonies on a regional basis rather than smaller institutions for each individual colony. It was hoped that specialist staff would be appointed to a new Colonial Research Service.^26^ The focus of attention for creating these regional organisations was initially East Africa, partly it seems because of calls by the Director of the East African Agricultural Research Institute at Amani, Dr A.G. Hill, for a new site for his station. Britain had inherited the agricultural unit at Amani after World War I when it was given the mandate for Tanganyika, previously a German colony. Amani had been allowed to fall into dereliction until the Colonial Office, shamed by the suggestion that this symbolised British neglect of agricultural research, had revived its fortunes during the mid-1920s.^27^ It was considered unsuitable for continuing research in agriculture, however, due to the shortage of land for experimental plots surrounding the hillside station. Hill also complained of the effects on staff morale of its extreme isolation, saying that the station had the atmosphere of a monastery.^28^

Detailed recommendations for new regional organisations were endorsed by the East African authorities at a conference on agricultural and forestry research in Nairobi in July 1947.^29^ It was agreed that there would be two new regional organisations in Kenya, both headed by researchers who worked in ARC units in Britain. The East African Agricultural and Forestry Research Organisation (EAAFRO) at Muguga in Kenya replaced the institute at Amani with Dr B.A. Keen of the ARC’s Rothamsted Experimental Station as its head. The East African Veterinary Research Organisation (EAVRO) was founded at Kabete under the direction of Dr E.G. White, a pathologist from the ARC’s Rowett Research Institute.^30^  In addition to these organisations in East Africa, the CCAAHFR also began making plans for the regional organisation of research in West Africa.^31^ Equivalent organisations to those in East Africa were never eventually created, but a West African Rice Research Station was opened in Sierra Leone in 1951. The West African Cocoa Research Institute and the West African Oil Palm Research Station also operated in the region, receiving funds from the research allocation.

The place of the regional research organisations in the plans of the CCAAHFR for colonial agriculture was explained in a document published in 1948.^32^ Here it was said that the chief objective was to ‘convince the scientific world that the colonial research worker is to have a fair deal’. In their recommendations, the CCAAHFR set out various conditions that it argued were necessary for efficient research, including satisfactory terms of service for scientific workers, the need to alleviate the isolation of scientific workers in the colonies, and the desirability of giving researchers reasonable freedom to determine their own research problems. The CCAAHFR argued that these conditions would be fulfilled in the new regional research establishments. The Secretary of the CCAAHFR, H.H. Storey, also emphasised the absolute necessity of placing each regional organisation under the control of a Director with research experience:

We doubt whether anyone who has not been an active research worker for a part of his life can effectively lead a research team with the understanding and appreciation that will bring out the best of which members are capable.^33^

In an earlier draft of the same document, Storey had been more explicit in his reasons why agricultural officers who were administrators in the Colonial Agricultural Service and did not possess research experience were not suitable individuals to supervise the work of agricultural researchers.

The normal administrator, with ideas based on command and orderliness, must find it difficult to accept a position as a leader of a group of individualists; and attempts to impose discipline or order on the research worker can be fatal to productive research.^34^

Arguments for the placing of responsibility for overseeing research into the hands of scientists who had themselves forged careers in research drew upon the independent status of the ARC’s research units in Britain for their power. The CCAAHFR stated that it wished to introduce to Britain’s colonies units for research similar to those that the ARC and MRC administered at home. In Britain agricultural research institutions such as the Rowett Research Institute were directed by researchers who reported to the ARC. The ARC, like the other research councils, was controlled by a committee composed of academic scientists who were in turn accountable to the Privy Council rather than a minister with departmental responsibilities. The significance of this arrangement was said to be the fact that the work of the units was determined by researchers with relevant experience and removed from the narrower interests of government departments.^35^ Similarly, in East Africa the plans were for regional research organisations that would be quite separate from the technical departments of the colonial governments, and would not be directed by these departments in terms of the work that was done.

With the creation of EAAFRO and EAVRO the CCAAHFR had achieved its goal of creating new regional laboratories headed by experienced agricultural researchers. Keen and White had responsibility for the content of the research that was done in their institutions, in communication with the CCAAHFR. They did not have to seek approval for their research plans from the Director of Agriculture employed by the governments of Kenya, Tanganyika or Uganda. The CCAAHFR expressed its desire to see the full support of colonial authorities for this arrangement.^36^ It was not entirely clear, however, that endorsement of this new apparatus for agricultural research would be forthcoming. Visits by members of the London committees to Africa and meetings with officers from the colonial administrations had made clear the objections of colonial governments to some of these plans. Generally, the complaint that was made was that colonial departments would not support research schemes that were forced upon them by metropolitan bodies.^37^ Accordingly, in its recommendations for the organisation of research, the CCAAHFR took great pains to emphasise that, whilst technical departments would not necessarily have the final say on the nature of research, co-operation with the new regional institutions would be sought. The CCAAHFR stated that it was a guiding principle for organising research that ‘the resulting knowledge obtained flows freely to those who will apply it’ and in the colonies it was the departments who were ‘users of research results’. In addition, the CCAAHFR said that it was important that research did not appear to those in the colonies as if it had been imposed from outside, and therefore it was inappropriate to have organisations that were entirely administered and directed from London. Research work, it said, should arise in response to local needs.^38^

Some degree of decentralisation when it came to organising agricultural research, with some responsibility devolved to bodies in Africa and attention paid to local demands, was entirely in keeping with Colonial Office policy from 1947 onwards. Under Arthur Creech-Jones the Colonial Office had moved away from its stance of the early 1940s in which it has sought direct interference in colonial affairs. This earlier approach had been prompted by frustration over the slow rate of progress in colonial development, attributed to the laissez-faire attitudes of the past, and inadequacies of the colonial administrations when it came to planning development.^39^ The new African Policy, largely created by the Head of the African Division, Andrew Cohen, had as its central aim the promotion of effective local government in the African territories. Local government would offer increasing responsibility for the running of local services for African peoples, as a preparation for eventual self-government. A general trend towards devolved responsibility meant that colonial governments would no longer be merely instructed by the Colonial Office with respect to new initiatives. Instead, progress would occur through a process of consultation and advice.^40^

The CCAAHFR intended that co-operation in the colonial territories would be achieved through the creation of regional councils for agriculture, animal health and forestry upon which would sit the Directors of EAAFRO and EAVRO, representatives of the colonial departments, agricultural producers and officers from local commodity research stations. This would allow the local agricultural departments to make recommendations for research. There were, however, limitations to how much local influence over research was acceptable to the CCAAHFR. The role of the regional councils was to be advisory rather than executive, ensuring that the Director of Research of EAAFRO and EAVRO was not in a subordinate position to any local Director of Agricultural Services (who was generally assumed to be an administrator with no research experience).^41^ The final authority in deciding the nature of research in EAAFRO and EAVRO was to be the Director of Research at the institution, supported in this task by the CCAAHFR in London. The achievement of the CCAAHFR was the creation of new research institutions that were slightly removed from the existing government machinery in the East African colonies.

The arrangements for research introduced by the CCAAHFR in East Africa after 1945 reconfigured the relationships amongst agricultural staff that worked in the region. Members of the existing Colonial Agricultural Service were officers who had contact with African farmers and instructed them in farming techniques. They were engaged by other members of the colonial administration to implement development policies such as soil erosion control or resettlement schemes. The increasingly large numbers of agricultural researchers working in institutions such as EAAFRO and EAVRO did not necessarily have any direct contact with African farmers but worked in relatively self-contained research institutions where they might be entirely involved in laboratory work such as developing vaccines. They had some independence from the Departments of Agriculture and the Colonial Administrative Service and a closer relationship than other agricultural officers with London-based committees. Agricultural expertise after 1940 therefore was more differentiated than many existing accounts of experts in Africa would currently indicate and importantly these individuals did not all have the same relationship with the colonial state and colonial peoples. Paying close attention to such differences in role is important if we are to produce more nuanced understandings of the role of agricultural expertise in the late colonial period. The creation of large organisations for agricultural and veterinary research after 1940 would seem to question the claim that one of the problems with expert-led interventions in Africa was a frequent absence of good understandings of the African environment.^42^ However, whilst agricultural research was better funded and organised after 1940 than at any previous point, the fact that research was slightly removed from agricultural practice raises new questions about the overall co-ordination of knowledge and action in Africa during this period.

## The Organisation of Colonial Medical Research

The CCAAHFR argued that the best way to greatly expand fundamental research in agriculture in the colonies was to create new institutions that would prove attractive to research staff by giving researchers the freedom to pursue their work without interruption. The approach of the CMRC was different. Rather than focus on the formation of new institutions in the colonies the CMRC insisted that medical research would flourish if the committee itself had a prominent role in determining the choice of research projects and the selection of medical research personnel. Control over the appointment of researchers was a priority for the CMRC in a way that it never was for the CCAAHFR. The premise was that the quality of medical research in Britain’s colonies was predicated on the quality of researchers, and only the MRC was in touch with medical researchers of merit. In addition, the direction of medical research activities in the colonies by the CMRC in London was considered essential. Medical research workers were said to only be willing to work on projects in which it was clear that the MRC was the authority as only the presence of members of the MRC could reassure researchers that the right conditions would be provided for their work. This effectively meant that colonial administrations, including members of the existing Colonial Medical Service, could not necessarily expect to have much say in the future direction of medical research.

At the initial meeting of the CMRC, A. Landsborough Thomson emphasised the importance of the principle that progress in colonial medical research would depend above all else on securing the services of the most able researchers, ‘It was important to begin by choosing suitable men and giving them considerable freedom in their choice of subjects.’^43^  From August 1946 the CMRC’s Personnel Sub-Committee interviewed potential candidates for research work and since the Colonial Research Service was not yet formally established, appointed researchers on temporary contracts.^44^ The result of the CMRC’s concern with the quality of research personnel was that staff deployed to work in colonial medical research during the second half of the 1940s were almost entirely hand-picked by the committee. The exception was two researchers already in the colonies who made applications to the CMRC for funding for their work. One was Dr W.S.S. Ladell, a biochemist at Yaba Medical School in Nigeria, who carried out a study into the effect of hot climates on the physiology of African labour.^45^ The other was Dr E.G. Holmes who received approval for funds of around 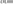 for a ten-year biochemical and physiological project at Makerere College, Uganda.^46^

In general, however, the CMRC claimed that the introduction of proper research would be achieved by the deployment of new workers to the colonies and not by encouraging existing members of the Colonial Medical Service to carry out more research. Mellanby was recorded as saying at a meeting of the CRC in 1942 that, ‘much of the research that was being done at present was ”jobbing” research and not what he described as the real thing. The problem seemed to be to superimpose the real thing on present institutions’.^47^ In the view of Mellanby the only real guarantee of the quality of individual selected for colonial research would be previous employment with the Medical Research Council in Britain informing officials that, ‘the Medical Research Council would welcome the chance to send their first class men out to the Colonies’.^48^ This disregard for the work of members of the existing Colonial Medical Service was expressed despite the fact that R.S.F. Hennessey, Assistant Medical Adviser to the Colonial Office, had prepared a list of 157 academic papers published by officers of the service to demonstrate to the CRC the significance of the contribution to medical research made by such individuals in the past.^49^

If the CMRC adhered to the principle that an expansion of high-quality medical research in Britain’s colonies would require an influx of new personnel who had been selected for this purpose by the committee itself, then the second principle that it promoted was that colonial medical research must be under the central direction of the CMRC in London. Research projects were generally suggested by individuals who sat on the committee rather than colonial medical departments. The focus of the schemes nominated by the CMRC was predominately on a list of nominated tropical diseases (Table 1).^50^  Two exceptions to an emphasis on curative medicine were a project that dealt with human nutrition, and the East African Medical Survey. Basic research into human nutrition was funded by the MRC and carried out at its Human Nutrition Research Unit. This was co-ordinated with a Field Research Station and Field Research Working Party in The Gambia supported by the Research Fund and overseen by a Nutrition Sub-Committee of the CMRC. The human nutrition project was conceived with the intention of relating data on the diet and general health of people in the Gambia with wider agricultural and socio-economic factors.^51^ However, there was some controversy as the project progressed over the extent to which the CMRC were willing to accept responsibility for the latter aspects of this work which were not considered to be ‘medical’.^52^ The East African Medical Survey was initially devised as a project that would study the African workers involved in the Groundnut Scheme in Tanganyika. When this original idea was abandoned the project was reformulated with a focus on the collection of statistical information on the health of village populations as a precursor for implementing effective preventative health care policies in rural areas.^53^

**Table 1:** Allocations from the research fund for medical research, by field, 1940–60. Source: Table of expenditure from J. Farley, *Bilharzia: A History of Imperial Tropical Medicine*, (Cambridge: Cambridge University Press, 1991), 275.

Research area	Allocation from research fund (  )
Trypanosome/tsetse	1,811,662
Viruses	762,129
Malaria	571,791
Nutrition	427,947
Physiology of hot climates	193,094
Filariasis	136,666
Trachoma	114,959
Immunity	110,995
Leprosy	56,429
Scrub typhus	56,257
Relapsing fever	49,390
Loasis	49,315
Bilharzia	48,828
Leishmania/kala azar	42,383
Typhoid	38,750
Tuberculosis	15,838
Flies/ticks	23,610
Guinea worm	7617
Onchocerciasis	2486
Trichinella	600
Total	4,520,296

The CMRC made arrangements between 1945 and 1950 to take over existing research institutions in the colonies and to set up a number of new units. It accepted the offer made by the Rockefeller Foundation to take on the virus research centres at Entebbe, Uganda and Lagos, Nigeria and it also accepted responsibility for a laboratory at Freetown in Sierra Leone that had belonged to the Liverpool School of Tropical Medicine.^54^ By 1952 the CMRC had also formed a Filariasis Research Unit in East Africa and an East African Malarial Unit.

Many investigations supervised by the CMRC had a component that consisted of laboratory experimentation and this was often done in research establishments in Britain such as the Liverpool and London schools of tropical medicine, the MRC’s own National Institute for Medical Research or the Lister Institute. In many cases, the MRC funded metropolitan work, leaving investigations that required fieldwork in the colonies to be financed using the research fund. The CMRC wished to see the movement of research staff between centres of tropical medicine in Britain and the colonies, and also movement between different locations in the Colonial Empire. Regular contact with metropolitan research organisations was said to be crucial in order to attract outstanding researchers. High-flying medical research workers were said to be in great demand and colonial research would only be able to attract these individuals if the arrangements for the administration of research fulfilled certain criteria. As well as the freedom for researchers to choose their own research projects, the CMRC identified the central direction of the research service by its own academic members as key. Central co-ordination of research by the committee in London would enable researchers to be in close contact with ‘the world of research’ which included ‘the general stream of research in the centres of learning at home’. In addition, the movement of research workers freely between colonies was necessary since ‘only by deploying research workers as opportunity offers can effective use be made of the limited resources of the first-class scientific manpower available’.^55^ The claim that mobility of personnel was desirable was only possible because of the definition by the CMRC of medical research problems as disease-based issues rather than issues that arose from particular communities or places. In general, the first consideration in creating a research project was a disease or treatment, rather than a specific colony or region, and this extraction of medical problems from their context made it possible to talk of the investigation of one issue, such as malaria, in a number of different colonies or metropolitan locations.

The fact that the CMRC chose and planned many of the research schemes to be done, personally selected research staff, and then directly administered and supervised much of this research amounted to a large degree of central direction by this body. This creation and direction of research projects by the London committees was not unusual and was a practice that the CCAAHFR, for example, was involved in at times through its sub-committees dealing with cocoa, fertilisers or stored products. The CMRC, however, dealt with a relatively small number of projects proposed by colonial administrations. In comparison to the CCAAHFR, the CMRC was reluctant to consider alternative arrangements for administering research schemes that might involve devolved responsibility to organisations in the colonies, or collaboration with local administrations. The CMRC noted in 1947 that it was aware that regional bodies to co-ordinate research were planned for agriculture and veterinary science in East Africa but:

the CMRC has considered that a different form of organisation would be preferable for medical research so as to keep medical research workers in East Africa in close contact with medical research in the UK and the rest of the Empire.^56^

The committee expressed the view that, whilst researchers should cultivate cordial relations with the colonial governments and observe their regulations for discipline, ‘in all research matters they will be outside the jurisdiction of the authorities of the Colonial Territory which they work’.^57^ One reason for this was ‘A general research organisation must be independent of local influence which, inevitably, would tend to divert it from its proper objects’.^58^

The key to successful medical research was, as it had been with agricultural research, freedom for high-calibre research staff to pursue their work without interference. The threat to long-term fundamental investigations came from members of the colonial administrations including existing scientific and medical officers who, it was claimed, had little experience of research. In the case of agricultural research, freedom of action had been achieved by the creation of large new research organisations that removed agricultural researchers from the control of the Departments of Agriculture. In the case of medical research, freedom of action was to be achieved less as a product of new research laboratories and more as the outcome of the complete authority over medical research that the CMRC sought from London. Medical research workers, who might move between different metropolitan and colonial locations, were accountable to the CMRC, and not to any colonial administration. This was desirable, it was claimed, as the MRC was the pre-eminent authority in the field of medical research and only its members could determine the conditions that medical researchers required to undertake their work. In addition, medical research in the colonies would only have sufficient cachet to attract the most able workers if it were clear to these individuals that they would effectively be working under the aegis of the MRC.

One outcome of the complete authority over medical research sought by the CMRC was the marginalisation of existing medical staff of the colonies when it came to the organisation of research during the 1940s. Individuals in the medical, tsetse and game departments of the East African colonies before World War II had hoped to create inter-colonial committees to deal with disease problems and to collate information on health that would operate on an East African basis. In 1936 a Standing Medical Research Committee was approved by the East African Governors and met in August of that year.^59^ The initiative in the development of new projects and bodies to explore questions of health was located in Kenya, Tanganyika and Uganda. A trajectory in which medical professionals and specialists such as entomologists based in East Africa would determine the future direction of medical research in the region was disturbed in 1945 with the emergence of the Colonial Medical Research Committee. Whilst the CCAAHFR had accepted the need for bodies in East Africa to co-ordinate research with agricultural practice at the local level to some extent, the CMRC was very slow to embrace similar arrangements for medicine. During the latter half of the 1940s this uncompromising approach of the CMRC brought it into increasing conflict with the Colonial Office.

## A Struggle for Authority in the Organisation of Medical Research

When it was faced with the issue of how to expand its research activities after 1940 the Colonial Office decided that the involvement of eminent scientists drawn from the research councils in Britain would be necessary in order to confer upon colonial research a sufficiently high status to attract good-quality research workers. The CCAAHFR and the CMRC were created with this object in mind, with representatives of the ARC and MRC appointed to these committees so as to allow the research council model to inform new arrangements for colonial research and to bring this work into closer contact with domestic institutions from which high-calibre research staff might be recruited. The relationship between the CCAAHFR and officials at the Colonial Office was very good. In contrast, relations between the CMRC and the Research Department of the Colonial Office became increasingly fraught during the 1940s.

Good relations with the MRC were considered by officials to be key if the Colonial Office was to achieve its aim of securing the services of British medical researchers for new projects in colonial research. In the years leading up to 1947, the Colonial Office concurred with many of the plans of the CMRC and the other research committees, including freedom of action for researchers, the creation of a new research service, and a degree of central direction in research. The Colonial Office believed that it had given the research committees a degree of autonomy that was unprecedented, considering that these committees were staffed by individuals who worked on a voluntary basis and were involved in the organisation of colonial research only through the invitation of the Office. It became increasingly clear during the course of the 1940s that not only was the CMRC unappreciative of the efforts of the Colonial Office to meet the requirements of the scientists it worked with, the committee could be openly hostile. It became apparent that the CMRC aimed to force the Colonial Office to relinquish control of funds for medical research in the colonies to the MRC.

The issue was that the MRC prided itself on the fact that it was not a body that submitted to any other authority when it came to matters of medical research. The first Secretary of the MRC, Walter Morley Fletcher, is said to have firmly believed that all funds for medical research should come under the purview of the MRC as it was the only body in Britain capable of judging the worth of research proposals in medicine. The council had a tendency to absorb other bodies in Britain concerned with medical research. In the inter-war period the MRC’s desire to be the pre-eminent authority in matters of medical research in Britain caused some friction between the MRC and other institutions, including the Ministry of Health and the Royal Colleges.^60^ Fletcher strongly resisted the idea that the MRC should be in any way subordinate to the Ministry of Health and a concordat was devised in 1924 in which the fields of responsibility of the two bodies were carefully demarcated.^61^ The CMRC, as an extension of the MRC, resisted any intervention by the Colonial Office that could be perceived as an attempt to place limits on its freedom and power to oversee colonial medical research. Such interference was considered necessary by the Colonial Office, however, because of the possible consequences for political stability in the colonies and the good relations between London and the territories of the Colonial Empire if the CMRC were left to ride rough shod over local sensitivities. As the 1940s progressed it became clear to officials that the particular circumstances of Britain’s colonies meant that arrangements and procedures developed for domestic research could not necessarily be directly transferred to the Colonial Empire. There was in fact a limit to the extent to which the research council model was appropriate for Britain’s colonies.

A number of issues served as flash points. One was the desire of the CMRC to appoint and deploy workers as it saw fit, freely moving researchers between colonies, and to metropolitan research institutions, at will. In practice, the recruitment and movement of researchers required co-operation and agreement between the Colonial Office, the Treasury and colonial governments. The CMRC failed to appreciate the nature of the relationship between the Colonial Office and the colonial governments in particular. The Colonial Office clarified its position in 1950:

It will be common ground that, in order to achieve the maximum of success, any investigation conducted in a Colony must have the good-will and co-operation of the Colonial administration concerned. The Secretary of State is not in a position to order any particular investigation to be carried out in any particular territory. The government of the territory has to be asked in the first place whether it agrees to the investigation being carried out and how soon.^62^

The CMRC would not acknowledge the necessity of a diplomatic approach by the Colonial Office when dealing with colonial governments, however, and accused the Office of inefficiency and inflexibility when it came to the deployment of new medical research staff.^63^  Apart from complaints that the Colonial Office was too slow in making appointments, the CMRC expressed hostility to the plans for the Colonial Research Service presented to it. The problem was that the CMRC had always envisaged a Colonial Medical Research and Laboratory Service that was independent of the other research services, and under the direct control of the CMRC.^64^ It was clear by 1947 that the Colonial Office was formulating a scheme to introduce one single Colonial Research Service that would encompass workers from all fields. The CMRC saw the combined research service as a threat to its freedom of action and its authority when it came to directing medical researchers.^65^ The solution proposed by the CMRC to the alleged shortcomings of the Colonial Office when it came to appointing research workers was to demand the office hand over the responsibility for the planned Colonial Research Service to the MRC on the grounds that this body had more experience of dealing with the special requirements of a research service.^66^ In early 1950 the CMRC went further, writing to the Colonial Office recommending that all funds for colonial medical research from the CDW Act should be devolved to the MRC.^67^

The arguments of the CMRC were almost entirely geared towards preserving the MRC’s ultimate authority over researchers, and therefore colonial medicine, without having to endure the scrutiny of either the Colonial Office or the Treasury for every decision it made. The CMRC, dominated by individuals closely associated by the MRC, if not appointed by this body, was adamant that medical research must in no way be influenced by the concerns of government departments, and it used the position of the MRC itself as an exemplar, referring to its relationship with another government department, the Ministry of Health.^68^ The problem was summed up by Charles Eastwood of the Colonial Office, who commented to the Treasury:

The Committee, as you will see, is an advisory body. But with the precedent before them of the independence of the MRC from the ordinary administrative machine in Whitehall, they have always taken the view that medical research policy must be controlled and administered solely by medical research men.^69^

This desire to ensure that it was scientists who had ultimate control of the research agenda and not administrators was also reflected in the arguments employed by the CCAAHFR to secure autonomy for researchers in East Africa from the colonial departments. However, whilst the CCAAHFR was most concerned with new forms of organisation for research within the colonies themselves, the CMRC was preoccupied with its own status, and that of the MRC, with respect to the Colonial Office.

The Colonial Office strenuously resisted the attempts of the CMRC to obtain complete control of medical research funded with the CDW allocation. Officials complained that the CMRC had proven to be a particularly uncompromising and uncooperative body of experts, something that was ascribed to the dominance of the committee by the MRC, and the personality of Mellanby.^70^ During the wrangles involving the CMRC and Colonial Office in 1950 Hibbert, Head of the Research Department, urged that the attempts of the CMRC to remove the field of colonial medical research from the sphere of influence of the Colonial Office must be fought ‘tooth and nail’. One of his criticisms of the CMRC was the very little first-hand experience of the colonies held by the members of this committee, the exceptions being Platt and Buxton.^71^

By 1950 it was clear that the desire of the CMRC to direct research in Africa from London, and its willingness to disregard the political conditions that existed in individual colonies, put it increasingly out of step with the trend in official policies and developments in the colonies themselves. In March 1950 the Colonial Office had produced its formal response to the demands that control of medical research for the colonies be transferred to the MRC. Apart from the constitutional reasons why the Secretary of State was unable to devolve his responsibilities for Colonial Development and Welfare to another body, the Colonial Office made it clear that it was not acceptable to impose medical research upon territories which were moving towards independence and where local peoples were increasingly represented on local legislatures. With regard to the native inhabitants of the colonies, ‘It is part of the process of education for self government that they should appreciate the value of research and they will not appreciate something in which they have no share’.^72^ Stronger words were used in private: Chief Medical Adviser of the Colonial Office, Dr E.D. Pridie, argued that total control of research by the MRC was unacceptable since, ‘the colonial peoples themselves would soon bitterly resent being considered as almost experimental animals in being subjected to research by outsiders without any control by their own government and their own medical service’.^73^ This assertion was borne out by the response of the African population of Tanganyika to the East African Medical Survey. Attempts by researchers to collate data on height and weight and to collect blood films from individuals had met with a great deal of resistance because of the intrusive nature of the researchers’ demands.^74^

Colonial Office attitudes towards the colonial administrations, and the people that lived under British rule, had undergone important changes since the beginning of the 1940s. The policies developed by Andrew Cohen now held that services within each colony should gradually be placed in the hands of African peoples operating through local government, and that relations with the colonies should be based on advice and consultation. Opposition to British rule produced violent unrest in the colonies: riots in the Gold Coast and the declaration of a state of emergency in Malaya in 1948. The views of the British medical establishment now seemed high handed at the least, and were perhaps even likely to cause serious dissent amongst colonial populations. The demands of the CMRC, therefore, that responsibility for colonial medicine be handed over to the MRC were quite unacceptable to the Colonial Office. As summed up by Hibbert:

practically all schemes of development and research have a political aspect, and the decision on any questions of policy or politics affecting the peoples of Colonial territories must rest with the Colonial Office administrative staff and the Colonial administrations.^75^

In order to effect some compromise with the CMRC, separate and more favourable scales of pay for medical workers based on those used by the MRC were introduced for the Colonial Research Service. The membership of the CMRC was altered, however, to include a greater proportion of individuals who were considered to support the Colonial Office view on research, such as individuals drawn from the Colonial Medical Service.^76^ By 1951 advisory bodies in West Africa and East Africa were planned to co-ordinate medical research with the requirements of each region.^77^ In 1953 the Standing Advisory Committee for West Africa became the West African Council for Medical Research with responsibility for supervising the work of the Virus Research Institute at Yaba, the Hot Climate Physiology Research Laboratory, the Leprosy Research Unit and the Loiasis Research Unit. The first meeting of the East African Standing Advisory Committee was held in March 1952 and an East African Council for Medical Research was formed from the East African Standing Committee in 1955. These new regional councils were endowed with considerable powers and moved steadily towards a position of autonomy during the 1950s.^78^

## Conclusion

The fact that the Colonial Office decided to appoint representatives of Britain’s research councils to oversee the spending of its research fund tells us something significant about the place of the research councils by the mid-twentieth century. The DSIR, ARC and MRC did not command the largest funds for research in Britain – more money was spent by the Service Departments; and yet it was these bodies that defined the very nature of research. They had achieved the position of pre-eminent authorities on the true nature of fundamental research and the conditions necessary for its successful prosecution. The high status of the ARC and MRC was predicated on the notion of freedom. The research councils, it was said, were not subordinate to the authority of civil servants and politicians and they ensured conditions for their researchers that served to encourage free enquiry. The development of the research council system in Britain was more than just the expansion of state funds for science, it was the establishment of a particular rhetoric about the nature of research and how it should be nurtured.

Whether or not officials at the Colonial Office subscribed to this liberal ideology of research, when it came to the expansion of colonial research after 1940 officials believed that explicit research council involvement would be essential for recruitment. A different type of scientist or medical worker was sought for work in the colonies after 1940 to those who had previously been recruited to the technical services. The Colonial Office wished to see expansion in numbers of high-calibre researchers – scientific specialists who most likely were currently working at a university or research unit in Britain and were in receipt of research council funds. The appointment of members of the ARC and MRC to new committees was intended to give the Colonial Office direct access to these individuals and it was hoped that the supervision of colonial research by committees such as the CCAAHFR and CMRC would give this activity the prestige it needed to be attractive to such workers. The CCAAHFR and CMRC were given a great deal of freedom in the early years of their work and set about organising research in agriculture and medicine with little interference from Colonial Office officials.

Both the CCAAHFR and the CMRC referred to the organisation of research at home by the research councils in their proposals for new apparatus for colonial agricultural and medical research. The notion that modelling colonial research on domestic arrangements was the most appropriate thing to do was not initially challenged by the Colonial Office, even though objections were raised by members of the technical departments in the colonies. Officials accepted the claim that the special nature of fundamental research meant that it required new arrangements. They also accepted the claim that no existing officer in the colonies was qualified to oversee this activity. What is striking here is the extent to which the activity of research was treated as something that had far greater cachet than other technical, scientific or medical work. The distinction that was made by the committees in London between fundamental research into general issues and short-term solutions of local problems was made concrete with the creation of separate research institutions in the colonies and a new Colonial Research Service. Scientific specialists employed for colonial research after 1940 and agricultural officers, doctors and veterinary officers did not necessarily work within the same colonial apparatus. The consequences and implications of the heterogeneous system of expertise this produced have not been examined by historians. We have yet to consider the legacy of the involvement of the ARC in creating semi-independent agricultural research organisations such as East African Agricultural and Forestry Research Organisation (EAFFRO). In addition there is the question of the significance of the emphasis placed by the MRC on the mobility of medical researchers in shaping the nature of colonial medical knowledge.

By the late 1940s officials at the Colonial Office were starting to raise some concerns about the organisation of colonial research and from 1947 there was an increasing desire to see better communication between those who were most in touch with the needs of the colonies and those who determined the research agenda. Of the two councils formed to deal with agricultural and medical research, the CCAAHFR displayed the more collaborative approach to dealing with the issue with its members acknowledging a role for agricultural officers in the colonial administrations in determining the direction of agricultural research. In the case of the CMRC, however, officials discovered that the appointment of a committee that contained many members of the MRC had given the MRC the opportunity to claim funds for colonial medical research for itself. The rhetoric that worked to elevate research and the research councils above other activities and bodies in the field of science and medicine was now being used to argue that only the MRC was qualified to administer Colonial Office research funds.

When it came to who was the final authority in matters related to the organisation of colonial research, the Colonial Office had asserted itself by 1950. Officials endorsed the principle of independence for research workers, as long as the actions of these workers did not appear to offend either the colonial administrations or the local populations of the colonies. It did not accept, however, that the specialist committees in London could be entirely left to take the initiative in organising colonial research. By 1950 Colonial Office officials believed that the expertise they possessed with respect to the social and political issues of the colonies was more valuable than any special expertise in research.

## Footnotes

1. BeckAnn, *History of the British Medical Administration of East Africa* (Cambridge, MA: Harvard University Press, 1970); Anna Crozier, *Practising Colonial Medicine: The Colonial Medical Service in British East Africa* (London: I.B. Tauris, 2007); Deborah J. Neill, *Networks in Tropical Medicine: Internationalism, Colonialism, and the Rise of a Medical Specialty, 1890–1930* (Stanford, CA: Stanford University Press, 2012); Douglas Melvin Haynes, *Imperial Medicine: Patrick Manson and the Conquest of Tropical Disease* (Philadelphia, PA: University of Pennsylvania Press, 2001).

2. TilleyHelen, *Africa as a Living Laboratory: Empire. Development and the Problem of Scientific Knowledge 1870–1950* (Chicago, IL: University of Chicago Press, 2011); Joseph Hodge, *Triumph of the Expert: Agrarian Doctrines of Development and the Legacies of British Colonialism* (Ohio: University of Ohio Press, 2007).

3. Hodge, *ibid.*; Christophe Bonneuil, ‘Development as Experiment: Science and State-building in Late Colonial and Postcolonial Africa, 1930–1970’, *Osiris*, 15, (2000), 258–81; see also the Special Issue of *Journal of African History* (2000), 41, 1.

4. ClarkeSabine, ‘A Technocratic Imperial State? The Colonial Office and Scientific Research, 1940–1960’, Twentieth Century British History, 18 (2007), 453–480

5. Beck, *op. cit.* (note 1).

6. For the notion of place-based knowledge as a useful term for the understandings that scientists had of particular locations, see Donna Mehos and Suzanne Moon, ‘The uses of portability: circulating experts in the technopolitics of Cold War and decolonization’, in Gabrielle Hecht (ed.), *Entangled Geographies: Empire and Technopolitics in the Global Cold War* (Cambridge, Mass: MIT Press, 2011), 43–74.

7. MayerAnna-K., ‘Reluctant Technocrats: Science Promotion in the Neglect-of-science Debate of 1916–1918’, History of Science, 43 (2005), 139–159; Frank M. Turner, ‘Public Science in Britain, 1980–1919’, *Isis*, 71 (1980), 589–608; Andrew Hull, ‘War of Words: The Public Science of the British Scientific Community and the Origins of the Department of Scientific and Industrial Research, 1914–1916’, *The British Journal for the History of Science*, 32 (1999), 461–81.

8. AlterPeter, *The Reluctant Patron; Science and the State in Britain, 1850–1920* (Oxford: Berg, 1987); Philip Gummett, *Scientists in Whitehall* (Manchester: Manchester University Press, 1980); J.B. Poole and K. Andrews, *The Government of Science in Britain* (London: Orion Publishing, 1972). Specifically on the DSIR see Ian Varcoe, ‘Scientists, Government and Organised Research in Great Britain, 1914–1916: The Early History of the DSIR,’ *Minerva*, 8 (1970), 192–216; R. MacLeod and E.K. Andrews, ‘The Origins of the DSIR: Reflections on Ideas and Men, 1915–1916’, *Public Administration*, 48 (1970), 23–45; Hull, -*ibid*

9. DeJagerTimothy, ‘Pure Science and Practical Interests: the Origins of the Agricultural Research Council, 1930–1937,’ *Minerva*, 31 (1993), 129–40; J. Austoker and L. Bryder (eds), *Historical**Perspectives on the Role of the MRC: Essays in the History of the Medical Research Council of the United Kingdom and its Predecessor, the Medical Research Committee, 1913–1953* (Oxford: Oxford University Press, 1989).

10. MellanbyEdward, ‘The state and medical research’, in *Harveian Oration at the Royal College of Physicians, 18th October 1938* (Edinburgh and London: Oliver and Boyd, 1939); A.V. Hill, ‘Science, national and international, and the basis of co-operation’, in *The Ethical Dilemma of Science* (Oxford: Oxford University Press, 1960), 23–33.

11. J. Austoker, ‘Walter Morley Fletcher and the Origins of a Basic Biomedical Research Policy’, in Austoker and Bryder, *op. cit.* (note 9).

12. Mellanby, *op. cit.* (note 10), 18–19.

13. EdgertonDavid, Science, Technology and British Industrial Decline, 1870–1970 (Cambridge: Cambridge University Press, 1996), 38

14. The allocation for research for the Medical Research Council for the years 1945 to 1950 was as follows: 1945–6, /article/back/fn-group/fn/p/mixed-citation/inline-formula ; 1946–7, /article/back/fn-group/fn/p/mixed-citation/inline-formula ; 1947–8, /article/back/fn-group/fn/p/mixed-citation/inline-formula ; 1948–9, /article/back/fn-group/fn/p/mixed-citation/inline-formula  and 1949–50, /article/back/fn-group/fn/p/mixed-citation/inline-formula  The allocation for research for the Agricultural Research Council for the same period was: 1945–46, /article/back/fn-group/fn/p/mixed-citation/inline-formula ; 1946-7, £300,000; 1947–8, £400,000; 1948–9, /article/back/fn-group/fn/p/mixed-citation/inline-formula  and 1949–50, /article/back/fn-group/fn/p/mixed-citation/inline-formula  The annual allocation for the research fund under the Colonial Development and Welfare Act of 1945 was /article/back/fn-group/fn/p/mixed-citation/inline-formula

15. ClarkeSabine, ‘“The chance to send their first class men out to the Colonies”: the making of the Colonial Research Service’, in Brett M. Bennett and Joseph M. Hodge (eds), Science and Empire: Knowledge and Networks of Science Across the British Empire, 1800–1970 (Basingstoke: Palgrave Macmillan, 2011), 187–208

16. TNA CO 847/13/13, Notes on a meeting between Lord Hailey and Malcolm MacDonald, 25 May 1939.

17. Clarke, *op. cit.* (note 4).

18. TNA CO 847/13/13, Draft memo to Treasury, 26 January 1939.

19. TNA CO 859/79/13, Note by J. Smith, 21 April 1941.

20. The Chairman of the CCAAHFR was the Secretary of the Agricultural Research Council (ARC), Sir John Fryer, with the Secretary of the Royal Society, Sir Edward Salisbury, as Vice-Chairman. The remaining members were Sir Joseph Barcroft (Director, ARC Unit of Animal Physiology at Cambridge), Dr J. Carmichael (Veterinary Research Division, May and Baker Ltd), Professor H.G. Champion (Professor of Forestry, Oxford University), Mr T. Dalling, Dr C.D. Darlington (Director, John Innes Horticultural Institute), Sir Frank Engledow (Drapers Professor of Agriculture, Cambridge), Professor J.W. Munro (Professor of Zoology and Applied Entomology, University of London), Professor J. Simonsen (Director of Research, Colonial Products Research Council), Professor J.A. Scott Watson (Chief Education and Advisory Officer, Ministry of Agriculture and Fisheries), Dr S.P. Wiltshire (Director, Imperial Mycological Institute), Mr J. Smith (Advisor to the Secretary of State for the Colonies on Animal Health), Dr H. Tempany (Agricultural Advisor), Dr W.A. Robertson (Forestry Advisor), with Dr H.H. Storey, a plant pathologist from the agricultural research station at Amani, Tanganyika, as secretary from October 1946; The National Archives (TNA) CO 908/1; TNA CO 908/2.

21. TNA CO 927/11/2. On the work of the Colonial Advisory Council of Agriculture, Animal Health and Forestry after 1940, see Joseph Hodge, ‘Science, Development and Empire. The Colonial Advisory Council on Agriculture and Animal Health’, *Journal of Imperial and Commonwealth History*, 30 (2002), 1–26.

22. TNA CO 927/3/4, ‘Regional organisation of research: Central Africa’, letter from Carstairs, 26 July 1946.

23. WorthingtonE.B., Science in Africa (Oxford: Oxford University Press, 1938), 23–24

24. *Ibid*

25. TNA CO 874/36/4, ‘Conference of African Governors. Papers’, AGC No. 26 ‘Note on the organisation of colonial research’.

26. TNA CO 908/1, CCAAHFR, Papers and minutes, 2nd meeting, 30 July 1945.

27. ChurchA.G., *East Africa* *A New Dominion. A Crucial Experiment in Tropical Development and its Signficance to the British Empire* (London: H.F. & G. Witherby. 1927), 95; Church said, ‘By neglecting to maintain this insitution in a state of efficiency, the local governments and the Imperial Government alike have given further evidence of appalling lack of appreciation of the function of scientific research in development.’; Major Sir Ralph Furse, *Aucuparius: Recollections of a Recruiting Officer* (Oxford: Oxford University Press, 1962), 143; TNA CO 927/12/3 gives some history of Amani; see also M. Worboys, ‘Science and Colonial Imperialism in the Development of the Colonial Empire, 1895–1940’ (unpublished PhD thesis: University of Sussex, 1979), 215 and 216.

28. TNA CO 908/1, CCAAHFR 1st meeting, 15 June 1945.

29. TNA CO 908/4, CAR(47)32, ‘Preliminary report on the Nairobi Conference, July 1947’.

30. *Colonial Research, 1947–1948*, Cmd 7493 (London: HMSO, 1948).

31. TNA CO 908/4, CAR(47)48, ‘Regional organization of agricultural research in West Africa’, 30 October 1947.

32. Drafts of Col. 219, the final version and CO comments upon it in TNA CO 927/88/5 and CO 927/88/6.

33. TNA CO 927/88/5, ‘Colonial Agricultural Research, General Policy’.

34. TNA CO 927/88/6, Agricultural Research Policy Sub-committee, 1945–7.

35. Mellanby, *op. cit.* (note 10); A.V. Hill, *The Ethical Dilemma of Science* (New York: The Rockefeller Institute Press, 1960), 49–50.

36. TNA CO 927/88/6, ‘Regional organisation of research’, 1947.

37. TNA CO 927/1/1; CO 900/2; CO 900/5.

38. Final version of Col.219 in TNA CO 927/88/5.

39. LeeJ.M. and PetterM., *The Colonial Office, War and Development Policy: Organisation and the Planning of a Metropolitan Initiative 1939–1945* (London: Maurice Temple Smith, 1982), 171; M. Havinden and D. Meredith, *Colonialism and Development: Britain and its Tropical Colonies, 1850–1960* (London: Routledge, 1993), 204–5.

40. HyamR.  (ed.), *The Labour Government and the End of Empire, 1945–1951*, Part I, Series A, Vol. 2 (London: HMSO, 1992), Introduction, xxiv–xxxi; R.D. Pearce, *The Turning Point in Africa, British Colonial Policy 1938–1948* (London: Frank Cass, 1982).

41. TNA CO 927/88/6.

42. For one of the oldest articles that stated that experts in Africa were often ignorant of local conditions see John McCracken, ‘Experts and Expertise in Colonial Malawi’, *African Affairs*, 81 (1982), 101–16. More recently, see Dorothy L. Hodgson, ‘Taking Stock: State Control, Ethnic Identity and Pastoralist Development in Tanganyika, 1948–1958’, *Journal of African History*, 41 (2000), 55–78. For an extremely interesting article that suggests that understandings of the Rufiji River Basin in Tanganyika during the colonial period were actually good see, Heather J. Hoag and May-Bitt Ohman, ‘Turning Water into Power: Debates over the Development of Tanzania’s Rufiji River Basin, 1920–1960’, *Technology and Culture*, 49, (2008), 624–51.10.1353/tech.0.006118831291

43. TNA CO 913/1. Landsborough Thomson had previously held the post of secretary of the MRC’s Tropical Medicine Research Committee, in operation between 1936 and 1939. It seems likely that his role at meetings of the CMRC was to give some continuity between the work of this earlier committee and the CMRC; *Colonial Research, 1945–1946*, Col No. 208 (London: HMSO, 1946).

44. TNA CO 913/1, 9th meeting CMRC, 21 August 1946.

45. TNA CO 913/1. Ladell issued regular progress reports to the CMRC. Ladell’s direct communications with the committee were a contravention of normal colonial service protocol. At times reports from Ladell arrived with the CMRC in London before they were formally dispatched from Lagos.

46. TNA CO 913/1, 9th meeting CMRC, 21 August 1946.

47. TNA CO 900/1.

48. TNA CO 900/1.

49. TNA CO 913/2.

50. TNA CO 913/1, ‘Notes on the future plans of colonial medical research’, 1945.

51. TNA CO 913/1, ‘Memorandum by the Nutrition Sub-Committee of the CMRC. Proposals for the establishment of a field working party in the Gambia’.

52. TNA CO 913/2, ‘Memorandum by the Nutrition Sub-Committee of the CMRC. Proposals for the establishment of a field working party in the Gambia’.

53. *Colonial Research 1946–1947*, Cmd 7151, 13; TNA CO 913/4.

54. TNA CO 913/1, The Rockefeller’s Laboratories in Freetown, Sierra Leone were taken over by the CMRC in 1949, *Colonial Research 1947–1948*, Cmd 7493.

55. TNA CO 927/175/1, ‘Colonial Medical Research Committee, Memorandum on the Organisation of Medical Research for the Colonies’, 1950.

56. TNA CO 913/2, ‘Medical research in central and East Africa’, 1947.

57. TNA CO 927/175/1, ‘Colonial Medical Research Committee, Memorandum on the Organisation of Medical Research for the Colonies’, 1950.

58. TNA CO 927/175/1, ‘Colonial Medical Research Committee, Memorandum on the Organisation of Medical Research for the Colonies’, 1950.

59. Beck, *op. cit.* (note 1).

60. Austoker and Bryder (eds), *op. cit.* (note 9). Carsten Timmermann and Helen Valier, ‘Clinical Trials and the Reorganization of Medical Research in Post-World War II Britain’, *Medical History*, 52 (2008), 493–510; Carsten Timmermann, ‘Clinical research in postwar Britain: the role of the Medical Research Council’, in C. Hannaway (ed.), *Biomedicine in the Twentieth Century* (Amsterdam: IOS Press, 2008), 231–54; Martin Edwards, *Control and the Therapeutic Trial: Rhetoric and Experimentation in Britain, 1918–1948* (Amsterdam: Rodopi, 2007).

61. Austoker, *op. cit.* (note 11).

62. TNA CO 927/175/1, ‘Organisation of medical research for the Colonies. Note by the CO’, March 1950.

63. TNA CO 913/2, ‘Memorandum on the organisation of research in and for the Colonies’, Eastwood, 23 June 1947.

64. TNA CO 913/1, 3rd meeting CMRC, 1 October 1945.

65. TNA CO 913/3, 23rd meeting CMRC, 24 November 1949.

66. TNA CO 913/2, ‘Memorandum on the organisation of research in and for the Colonies’, Eastwood, 23/6/1947.

67. TNA CO 927/175/1, ‘Organisation of medical research for the Colonies. Note by the CO’, March 1950.

68. TNA CO 927/175/1, ‘Organisation of medical research for the Colonies. Note by the CO’, March 1950.

69. TNA CO 927/175/1, Colonial Medical Research. General Policy, letter Eastwood to H. Clough, 1 June 1950.

70. TNA CO 927/175/1, Note from Hibbert to Eastwood, 18 November 1949.

71. TNA CO 927/175/1, Note from Hibbert to Eastwood, 3 March 1950.

72. TNA CO 927/175/1, Note by Pridie to Eastwood, 29 March 1950.

73. TNA CO 927/175/1, Note by Pridie to Eastwood, 29 March 1950.

74. HoldenPat, ‘Doctors and Other Medical Personnel in the Public Health Services in Africa, 1930–1965. Uganda, Tanganyika/Tanzania, Nigeria’, *Oxford Development Records Project* (1986), 49–50.

75. TNA CO 927/14/1, ‘Reflections upon the African Governors’ Conference Paper No. 20’, 12 November 1947.

76. TNA CO 927/175/6, 27th meeting CMRC, 20 November 1951.

77. TNA CO 927/175/6, 26th meeting CMRC, 15 May 1951.

78. *Colonial Research, 1952–1953*, Cmd 8971 (London: HMSO, 1953); *Colonial Research, 1955–1956*, Cmnd 52 (London: HMSO, 1956).

